# A Derived Network-Based Interferon-Related Signature of Human Macrophages Responding to *Mycobacterium tuberculosis*

**DOI:** 10.1155/2014/713071

**Published:** 2014-08-31

**Authors:** Kang Wu, Hai Fang, Liang-Dong Lyu, Douglas B. Lowrie, Ka-Wing Wong, Xiao-Yong Fan

**Affiliations:** ^1^Shanghai Public Health Clinical Center, Fudan University, 2901 Caolang Road, Shanghai 201508, China; ^2^Key Laboratory of Medical Molecular Virology of MOE/MOH, Shanghai Medical College, Fudan University, 138 Yixueyuan Road, Shanghai 200032, China; ^3^State Key Laboratory of Medical Genomics, Ruijin Hospital Affiliated to Shanghai Jiao Tong University School of Medicine, 197 Ruijin Road II, Shanghai 200025, China

## Abstract

Network analysis of transcriptional signature typically relies on direct interaction between two highly expressed genes. However, this approach misses indirect and biological relevant interactions through a third factor (hub). Here we determine whether a hub-based network analysis can select an improved signature subset that correlates with a biological change in a stronger manner than the original signature. We have previously reported an interferon-related transcriptional signature (THP1r2*Mtb*-induced) from *Mycobacterium tuberculosis* (*M. tb*)-infected THP-1 human macrophage. We selected hub-connected THP1r2*Mtb*-induced genes into the refined network signature T*Mtb*-iNet and grouped the excluded genes into the excluded signature T*Mtb*-iEx. T*Mtb*-iNet retained the enrichment of binding sites of interferon-related transcription factors and contained relatively more interferon-related interacting genes when compared to THP1r2*Mtb*-induced signature. T*Mtb*-iNet correlated as strongly as THP1r2*Mtb*-induced signature on a public transcriptional dataset of patients with pulmonary tuberculosis (PTB). T*Mtb*-iNet correlated more strongly in CD4^+^ and CD8^+^ T cells from PTB patients than THP1r2*Mtb*-induced signature and T*Mtb*-iEx. When T*Mtb*-iNet was applied to data during clinical therapy of tuberculosis, it resulted in the most pronounced response and the weakest correlation. Correlation on dataset from patients with AIDS or malaria was stronger for T*Mtb*-iNet, indicating an involvement of T*Mtb*-iNet in these chronic human infections. Collectively, the significance of this work is twofold: (1) we disseminate a hub-based approach in generating a biologically meaningful and clinically useful signature; (2) using this approach we introduce a new network-based signature and demonstrate its promising applications in understanding host responses to infections.

## 1. Introduction

It has been estimated that* Mycobacterium tuberculosis (M. tb)* infects as many as 2 billion people in the world. Between 5 and 10% of these infected individuals will likely develop active tuberculosis (TB) during their lifetime [1]. Approximately 1.4 million people each year die from TB [1].* M. tb *infection results in disease after immune cells fail to contain bacterial replication. As* M. tb* primarily resides within macrophages after being inhaled there is an urgent need to understand how the host macrophages respond protectively and pathologically to* M. tb* infection. Transcriptional profiling of host cell responses is an unbiased whole-genome approach that has already been applied to the whole blood [2–4] and blood cell subpopulations [2, 5] of TB patients and to the human macrophage cell line model THP-1 infected with* M. tb* [6]. The information-rich data obtained from these analyses hold great promise for exploring mechanisms of pathogenicity and immunity, for TB diagnosis/prognosis, and have potential implications for development of new TB vaccines.

Changes in the transcriptome cause changes in cell functions. Yet the changes in transcriptome and the resultant changes in cell function are generally mediated by changes in the availability of RNA sequences and proteins that function within cascades of network interactions. Working from the principle that genes do not function alone but in the context of networks, network-based interpretations of “omics” data can uncover novel insights for biomedical research [7–9]. In such a view, it would be of greater biological relevance if “omics” data were trained in the context of protein-protein interactions [3, 9, 10]. As a precedent, candidate genes identified from a RNAi functional screen for host genes important for regulating* M. tb *survival in macrophages have been analyzed in the context of protein-protein interaction data. This analysis revealed a pivotal role of the regulation of autophagy for survival of* M. tb* [11]. This kind of network-based approach has also been applied in other contexts, such as an AIDS-relevant network in macaques for predicting the magnitude of specific T-cell responses and viral loads [9] and a putative network underlying early human organogenesis [12].

Network analysis of highly expressed genes typically relies on preexisting knowledge of a direct interaction between pairs of highly expressed genes. However, expression of multiple genes often indicates interaction with a hub or a factor that interacts/associates with many other gene products. Connections via a hub can be missed by network analysis that is based solely on direct interaction between two expression-active gene products. As these connections are biologically relevant, we proposed that hubs could be exploited for creating a biologically relevant subnetwork of expression-active genes.

Recently, we have reported transcriptome analysis of human macrophage cell line THP-1 infected by different* M. tb* W-Beijing strains and have identified a core interferon-related transcriptional signature [6]. This core host transcriptional response seemed to be positively correlated with* in vivo* transcriptome data from patients with active pulmonary tuberculosis (PTB) and to some extent this signature decreased following clinical therapy of PTB [6]. Here, by reanalyzing our previously reported interferon-related signature with a new hub-based network analysis strategy, we aimed to produce a refined signature that was biologically and clinically more correlative with PTB patients. Interestingly, the new signature also showed greater correlation with patients with acquired immunodeficiency syndrome (AIDS) and malaria but not with patients with several other infections or inflammatory conditions. We propose that the improved interferon-related signature can be an attractive alternative to the established large interferon-related signature and should be more accessible to TB investigators interested in host cell response research.

## 2. Methods

### 2.1. Protein Interaction Network Data

The protein interaction information used in this study was obtained from the STRING database [16]. STRING contains both physical and functional interactions between proteins in a variety of organisms. We extracted these interactions from the human specific network where there was a combined score of at least 0.7. This criterion ensured high coverage without compromising data quality [16].

### 2.2. Derivation of a Network-Based Signature from Original THP1r2*Mtb*-Induced Signature

We first identified a number of genes (referred to herein as hubs) that made a minimum *i* number of direct connections in the STRING database with genes in our previously identified active interferon-related signature (THP1r2*Mtb*-induced) [12]. We then used this set of hubs to select all the interacting genes in THP1r2*Mtb*-induced signature and grouped them into a new subset known as THP1r2*Mtb*-iNet[*i*]. Genes in THP1r2*Mtb*-induced signature excluded from THP1r2*Mtb*-iNet[*i*] were then grouped into THP1r2*Mtb*-iEx[*i*]. We then assessed the biological relevance of each subset by its aggregate *z*-score [17]. The calculation of aggregate *z*-score was similar to that described in the original paper [13]. In general, the *z*-score of an individual gene was calculated from the significance (adjusted *p*) of the change in gene expression by subtracting it from 1 (see our previous work for the adjusted *p* values for 4 h versus 18 h after infection [6]) and this was then divided by the normal cumulative distribution function (CDF). Then the aggregate *z*-score was calculated as the summation of *z*-scores from genes in a subset divided by the square root of the number of genes in a subset. In essence, the aggregate *z*-score reflected the expression levels of a signature and allowed comparison of putative signatures with different numbers of genes. The higher the aggregate *z*-score of a signature was, the more transcriptionally active the signature was. The signature with the highest aggregate *z*-score was visualized using Cytoscape [18].

### 2.3. Enrichment Analysis of Transcription Factor Binding Sites (TFBSs)

PRomoter Integration in Microarray Analysis (PRIMA) was applied for TFBS enrichment analysis for genes in the derived signatures [14]. The analysis was based on the promoter region spanning from 2,000 bp upstream to 200 bp downstream of transcription start sites, using the entire EntrezGenes as testing background. Enrichments with Bonferroni-corrected *P* value < 0.01 were declared as significant.

### 2.4. KEGG Pathway Enrichment Analysis

The analysis was done in the web-accessible Database for Annotation, Visualization and Integrated Discovery (DAVID) v6.7, based on Benjamini and Hochberg-derived False Discovery Rate (FDR) [17].

### 2.5. Gene Set Enrichment Analysis (GSEA) against Transcriptomes from Patients with PTB or Other Diseases

GSEA is a nonparameter method for determining whether signature genes are overrepresented at the top or bottom of a predefined list of ranked genes (genes are ranked from high to low according to their expression levels) [19]. The list of ranked genes was predefined according to the available transcriptome data. A total of nine transcriptome datasets were retrieved for GSEA analysis from NCBI GEO with accession numbers GSE19491 [2], GSE31348 [20], GSE6269 [21], GSE11907 [15], GSE4124 [22], GSE6740 [23], GSE5418 [24], GSE40184 [25], and GSE7123 [26]. Among these publicly available datasets, we chose the first two (GSE19491 and GSE31348) datasets to determine the correlation of our signatures because both datasets contained transcriptome data that compare PTB against latent tuberculosis (LTB) or healthy control (HC) and followed the course of PTB therapy. In particular, GSE19491 contains whole blood transcriptome data from a large number of PTB patients, LTB patients, and HC recruited from London in the UK and Cape Town in South Africa. These samples were grouped into 5 cohorts: (1) training set, London volunteers with PTB, LTB, and healthy controls; (2) test set, London volunteers with PTB, LTB, and healthy controls; (3) validation set, Cape Town volunteers with PTB and LTB; (4) Test_set_seperated, neutrophils (Neut), monocytes (Mono), CD4^+^ T cells (CD4), and CD8^+^ T cells (CD8) separated from the blood of the test set PTB patients and healthy controls; and (5) longitudinal study, patients after 2 months (PTB_2 m) and 12 months (PTB_12 m) of treatment and healthy controls [2]. GSE31348 contains whole blood transcriptome data from PTB patients in Cape Town, South Africa, at diagnosis (before drug treatment, week 0) and at 1, 2, 4, and 26 weeks of treatment [20].

Other datasets involving patients with other infections or inflammatory conditions were included in our analyses to determine the specificity of our network-derived gene signature. GSE6269 contains transcriptome data of peripheral blood mononuclear cells (PBMCs) from young patients. In these young patients, the infecting pathogens were (1)* Escherichia coli*, (2) influenza A, (3)* Staphylococcus aureus*, or (4)* Streptococcus pneumonia* [21]. GSE11907 contains transcriptome data of PBMCs from patients with one of the following conditions: (1)* E. coli* infection; (2) systemic juvenile idiopathic arthritis; (3) systemic lupus erythematosus; (4) liver-transplant recipient undergoing immunosuppressive therapy; (5) metastatic melanoma; (6) type I diabetes; and (7)* Staphylococcus aureus *infection [15]. GSE4124 contains transcriptome data of PBMCs from HIV-1 positive/negative mothers with infants in Botswana, Africa. These mothers could be divided into the following three categories: (1) HIV-1 negative mother; (2) HIV-1 positive mother who perinatally transmitted the virus to her infant; and (3) HIV-1 positive mother who did not transmit the virus to her infant [22]. For GSE6740, CD4^+^ or CD8^+^ T cells were purified from four groups of participants: Group 1: HIV-1-negative volunteers; Group 2: individuals with HIV-1 infection within 6 months of study and asymptomatic when blood was drawn (acute HIV); Group 3: individuals with chronic progressive HIV-1 infection for at least 1 year and asymptomatic (chronic HIV); and Group 4: nonprogressor individuals with HIV-1 infection for at least 3 years (nonprogressor HIV) [23]. GSE5418 contains two groups of donors: one group is malaria patients from Cameroon, West Africa, where blood was obtained for PBMCs separation before and after chloroquine treatment; the other group includes healthy individuals from the USA who were experimentally challenged with malaria-infected mosquitos. PBMCs were obtained from these subjects before mosquito challenge and when a single parasite was identified by blood smear microscopy [24]. GSE40184 contains transcriptome data of PBMCs from treatment-naïve chronic hepatitis C virus- (HCV-) infected patients or healthy controls [25]. GSE7123 contains transcriptome data of PBMCs from African-American/black (AA) or Caucasian-American/white (CA) patients with chronic HCV infection and undergoing therapy with pegylated interferon-2a (peginterferon). Treatment doses were 180 *μ*g weekly by self-administered subcutaneous injection and ribavirin orally in a dose of 1,000 or 1,200 mg daily based on body weight of less than 75 kg or equal to or greater than 75 kg. PBMCs were separated from patients prior to therapy (day 0) and on days 1 (after injection of peginterferon), 2, 7, 14, and 28. In addition, these patients were divided into three categories based on their change in HCV levels as detected by a quantitative PCR-based assay: (1) marked, defined as a decrease in virus RNA levels of more than 3.5 log_10_ IU/mL on day 28 relative to baseline; (2) intermediate, decrease of 1.4 to 3.5 log_10_ IU/mL; and (3) poor, decrease of less than 1.4 log_10_ IU/mL [26].

GSEA results are reported as normalized enrichment score (NES) and FDR. A gene signature with a positive score is overrepresented at the top of a ranked gene list and indicates a positive correlation (upregulated expression) in the gene list, whereas a gene signature with a negative NES is underrepresented at the bottom of a ranked gene list and indicates the negative correlation (downregulated expression) in the gene list. An FDR of 0.05 or less indicates statistical significance of NES [19].

## 3. Results

### 3.1. Derivation of an Integrated Signature Capturing Essential Characteristics of a Previously Identified Interferon-Related Signature

**Figure 1:**
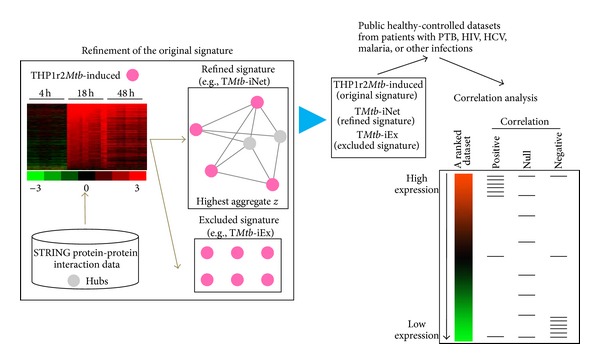
A network-based strategy to refine an expression signature. The expression signature of THP-1 macrophages in response to* Mtb* infection (THP1r2*Mtb*-induced) was used in this study [6]. To extract expression-active genes (pink dots) that are functionally linked genes, STRING protein-protein interaction data were used to select expression-active genes that interacted directly among themselves or interacted indirectly via a third protein that was defined thereby as a hub (grey dots). The degree of a hub corresponds to the number of direct interactions the hub makes. In the refined signature example, one hub has a degree of 4 and another has a degree of 3; thus the minimum degree of hubs in the example signature is 3. The selection of the minimum degree of hubs determines how transcriptionally active the refined signature will be. The final refined set was the most transcriptionally active one (i.e., T*Mtb*-iNet, which is refined based on hubs with minimum degree 14). T*Mtb*-iNet and the original signature (i.e., THP1r2*Mtb*-induced) as well as the cognate excluded genes (i.e., T*Mtb*-Ex) were then analyzed for their correlations with other patient-derived transcriptome datasets by GSEA.

**Figure 2:**
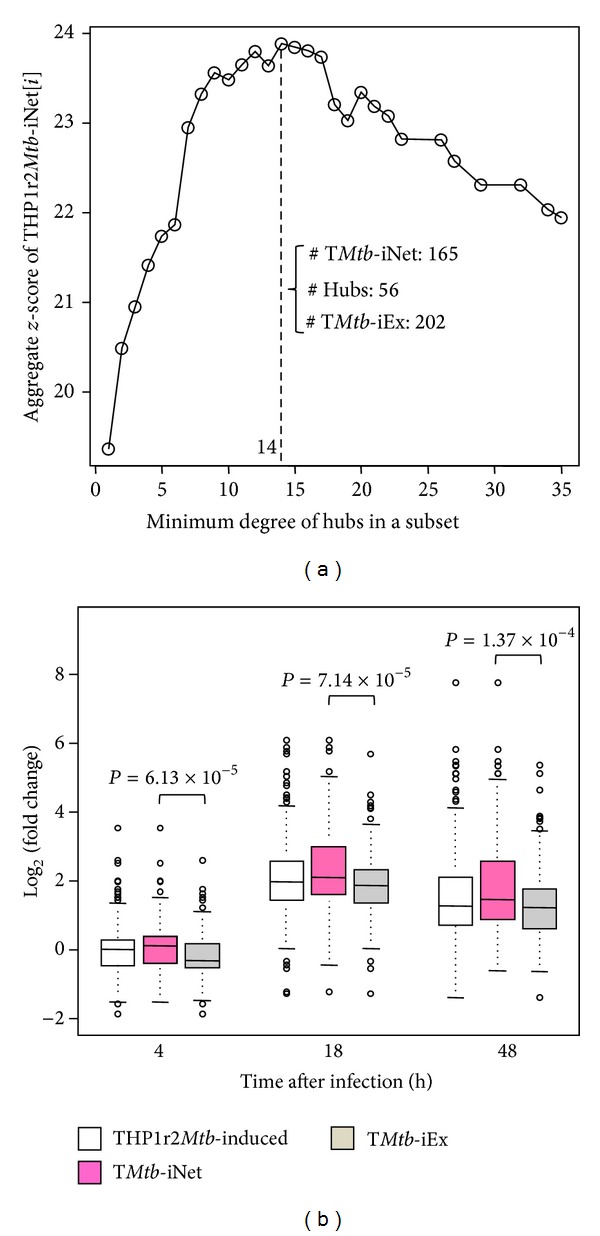
Aggregate* z*-scores of THP1r2*Mtb*-iNet[*i*] and box-plotting. Sets of integrated THP1r2*Mtb*-iNet[*i*] were extracted with involvement of different sets of hubs with minimum degree *i*. The adjusted *P* values of genes in THP1r2*Mtb*-iNet[*i*] at 18 h after infection relative to 4 h after infection were converted into *z*-scores. The aggregate *z*-scores of THP1r2*Mtb*-iNet[*i*] as a whole were then calculated, which enabled comparison among signatures with different sizes [13]. The sizes of the relevant signatures based on hubs with minimum degree 14 were also enumerated (a). Then the signature with the highest aggregate *z*-score (T*Mtb*-iNet), the cognate excluded signature (T*Mtb*-iEx), and the parent signature (THP1r2*Mtb*-induced) were box-plotted for mutual comparison of their expressions (b).

Previously, we identified an active interferon-related signature (THP1r2*Mtb*-induced) as a common transcriptional response of THP-1 cells to infection by different* M. tb* W-Beijing strains [6]. Since gene products function within the context of networks and perturbation of such networks often changes cell phenotype [8], we reasoned that the transcriptional core response could be better described/refined when integrated with protein-protein interaction data. To this end, we combined the previous data with protein-protein interaction network data to refine the original signature (Figure 1). We sought to identify which of the genes that showed a dominant expression pattern during* M. tb* infection were also highly linked among themselves or via a hub in the human interaction/association network. Then, the highly linked signature and the excluded signature, as well as the original THP1r2*Mtb*-induced signature, were subject to gene set enrichment analysis (GSEA) on publicly available patient-derived transcriptome data for validation and comparison (Figure 1). A hub was selected from the STRING protein interaction database based on the biological relevance of the hub, which was defined by the number of direct interactions (referred to herein as the degree of interaction) the hub made with expression-active gene products (i.e., gene products of THP1r2*Mtb*-induced). We grouped together all highly expressed genes, whose gene products mutually interacted directly or interacted indirectly via at least one of the hubs that had a minimum of *i* degrees of interaction, into the refined subset signature THP1r2*Mtb*-iNet[*i*]. As the minimum degree required for inclusion of hubs in a subset was increased, the number of hubs (Figure S1A) and the total number of interactions in the subset decreased dramatically (Figure S1B) (Supplementary Material is available online at http://dx.doi.org/10.1155/2014/713071). Also, more highly expressed genes were excluded from the new subset (Figure S1C). These excluded highly expressed genes were grouped into THP1r2*Mtb*-iEx[*i*]. The expression level of each THP1r2*Mtb*-iNet[*i*] as a whole was then assessed by aggregate *z*-score. This score allows comparison among gene groups with different sizes. The higher the aggregate *z*-score, the higher level of expression of THP1r2*Mtb*-iNet[*i*].  Figure 2(a) displays the distribution of aggregate *z*-scores as a function of minimum degree of hubs. The aggregate *z*-score for THP1r2*Mtb*-iNet[*i*] reached the highest when hubs had at least 14 degrees. We refer to the signature with 14 minimum degrees as T*Mtb*-iNet and the cognate excluded signature as T*Mtb*-iEx.  Figure 2(b) indicated that the T*Mtb*-iNet genes were expressed at significantly higher levels than the T*Mtb*-iEx genes.

**Figure 3:**
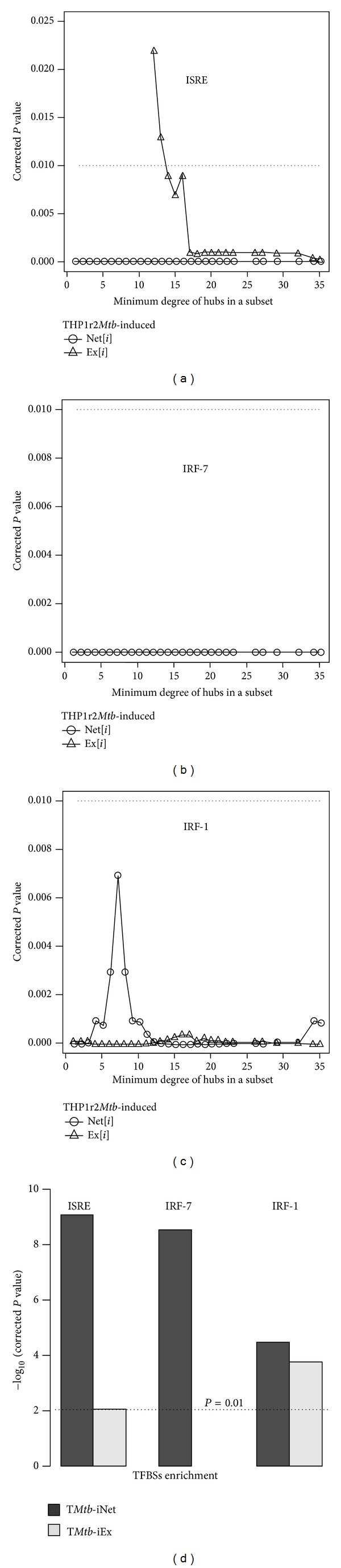
TFBS enrichment profiles of THP1r2*Mtb*-iNet[*i*]. Putative enrichment of TFBSs (i.e., ISRE (a), IRF-7 (b), and IRF-1 (c)) in the promoter regions of THP1r2*Mtb*-iNet[*i*] and THP1r2*Mtb*-iEx[*i*] (2,000 bp upstream to 200 bp downstream of transcription start site) was analyzed with Expander [14]. (d) The TFBS enrichment in T*Mtb*-iNet and T*Mtb*-iEx. See Table S2 for detailed Bonferroni-corrected *P* values.

**Figure 4:**
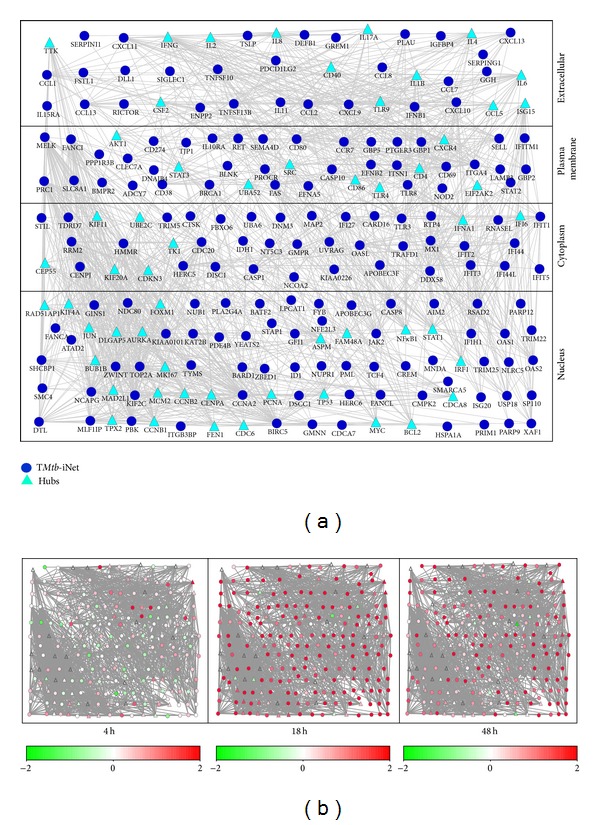
Illustration of T*Mtb*-iNet in the context of the molecular network. (a) T*Mtb*-iNet and its cognate hubs are laid out according to the information about subcellular localization as annotated in NCBI GO. Those nodes shown as circles are genes from T*Mtb*-iNet and those nodes shown as triangles are hubs with direct interaction with gene products in T*Mtb*-iNet. See the complete gene list in Table S2. (b) Illustration of the expression pattern of T*Mtb*-iNet as well as its cognate hubs in THP-1 cells at 4 h, 18 h, and 48 h after* M. tb* infection. See also Table S3 for detailed expression levels.

In our previous study, we showed that the promoter regions of genes in THP1r2*Mtb*-induced signature are significantly enriched for transcription factor binding sites (TFBSs) of interferon-related regulators (i.e., ISRE, IRF-7, and IRF-1) [6]. To validate that T*Mtb*-iNet genes were still representative of THP1r2*Mtb*-induced signature, we also looked for significant enrichment of these three putative TFBSs. Regardless of which minimum degree of hubs was utilized, we always observed the superior enrichment of ISRE and IRF-7 in promoter regions of genes in THP1r2*Mtb*-iNet[*i*] compared to genes in THP1r2*Mtb*-iEx[*i*] (corrected *P* < 0.05) (Figures 3(a) and 4(b)). In contrast, IRF-1 was significantly enriched in promoter regions of genes in both THP1r2*Mtb*-iNet[*i*] and THP1r2*Mtb*-iEx[*i*] (corrected *P* < 0.05) independent of the minimum degree of hubs (Figure 3(c)). We especially noted that the TFBS of IRF-7 was exclusively enriched in promoter regions of genes in THP1r2*Mtb*-iNet[*i*] but not of genes in THP1r2*Mtb*-iEx[*i*] (Figure 3(b)).  Figure 3(d) illustrates the consistent and superior significant enrichment of TFBSs in the promoter regions of genes in T*Mtb*-iNet compared to genes in T*Mtb*-iEx, derived using hubs with minimum degree 14.

**Figure 5:**
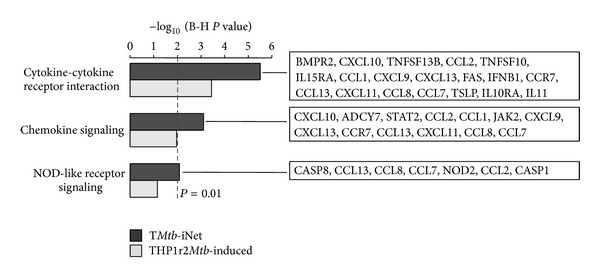
KEGG pathway analysis of T*Mtb*-iNet and THP1r2*Mtb*-induced signature. KEGG pathways enriched in the two signatures (T*Mtb*-iNet and THP1r2*Mtb*-induced) are displayed (FDR < 0.01). The refined genes in T*Mtb*-iNet are listed on the right. Also see the gene list in Table S5.

Figure 4(a) illustrates the layout of T*Mtb*-iNet plus its cognate hubs with minimum degree 14 according to the subcellular localization of their gene products. In this layout, the expression changes of all genes were color-coded, showing the overwhelming induction (upregulation) especially at 18 h after* M. tb* infection (Figure 4(b)). T*Mtb*-iNet significantly enriched the pathways of cytokine-cytokine receptor interaction, chemokine signaling, and NOD-like receptor signaling compared to THP1r2*Mtb*-induced signature (Figure 5 and Table S5). By contrast, T*Mtb*-iEx did not enrich any pathway.

In summary, by utilizing hubs with minimum degree 14, we obtained the network-based signature of T*Mtb*-iNet that displayed the highest expression significance (the highest aggregate *z*-score), without losing the enrichment of interferon-related TFBSs of ISRE, IRF-7, and IRF-1 in their promoter regions.

### 3.2. T*Mtb*-iNet Contains more Interferon-Related Genes than T*Mtb*-iEx

**Figure 6:**
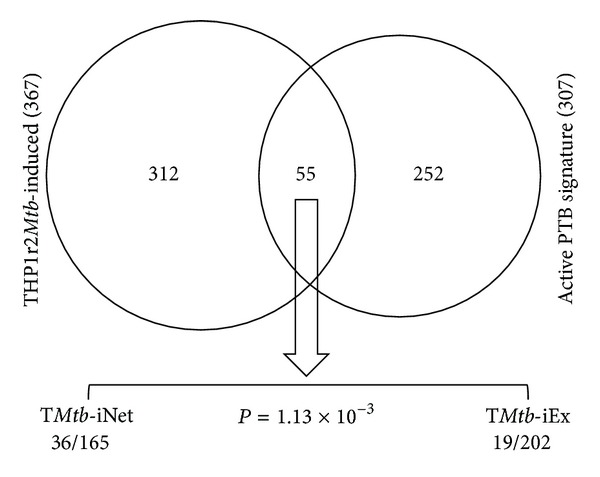
T*Mtb*-iNet preserved more genes overlapped with those in the active PTB signature. Of the 55 genes overlapped between the THP1r2*Mtb*-induced and active PTB signatures, 36 were still present in T*Mtb*-iNet, whereas only 19 were in T*Mtb*-iEx. This biased distribution is statistically significant (*P* = 1.13 × 10^−3^, Fisher's exact test).

**Figure 7:**
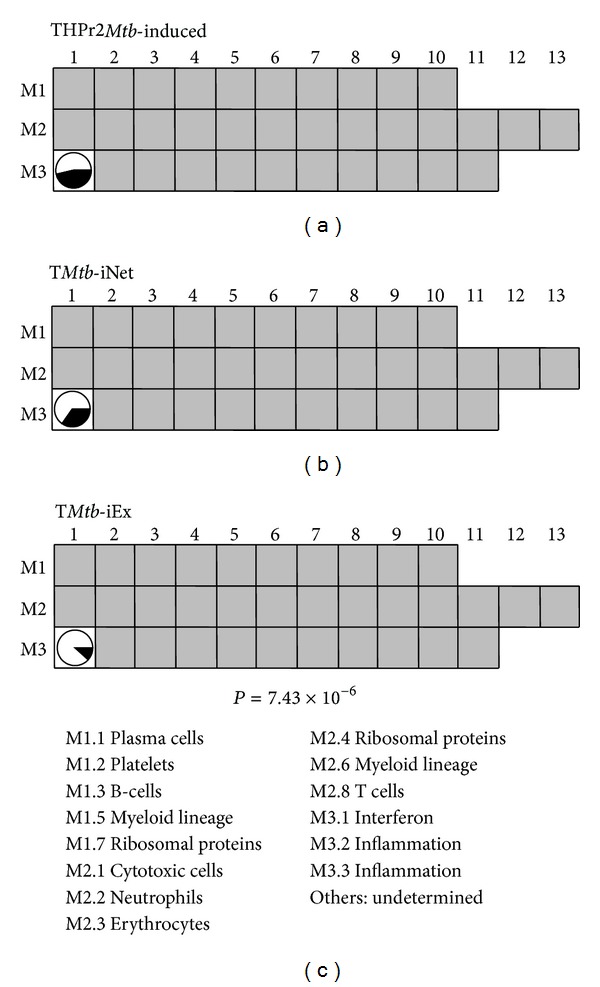
T*Mtb*-iNet overlapped more genes with an interferon-related module. The interferon-related module (M3.1) contained 94 genes, almost half of which (44/94) were present in the THP1r2*Mtb*-induced signature (depicted as the proportion of black in the circle at position M3.1). Of these 44 genes, 33 were also present in T*Mtb*-iNet, whereas only 11 were in T*Mtb*-iEx. This biased distribution is statistically significant (*P* = 7.43 × 10^−6^, Fisher's exact test). Functional clarification of each module is indicated in the lower panel [15].

Interferon-related genes are expected to function in the context of interferon-relevant molecular networks. Since THP1r2*Mtb*-induced signature correlates with interferon-related processes, we determined whether T*Mtb*-iNet contained more interferon-related genes than T*Mtb*-iEx did. Based on transcriptional profiling of whole blood from a large number of pulmonary tuberculosis (PTB) or latent tuberculosis (LTB) patients and healthy volunteers, Berry et al. reported a PTB specific interferon-inducible neutrophil-driven blood transcriptional signature (393 transcripts representing 307 unique Entrez Genes) compared to LTB and healthy controls [2]. We reported earlier that 55 of these signature genes were significantly (*P* < 10^−5^) present in the THP1r2*Mtb*-induced signature [6]. We found that 36 of the 55 overlapped genes were also present in T*Mtb*-iNet, whereas only 19 were present in T*Mtb*-iEx (*P* = 2.65 × 10^−4^) (Figure 6). Chaussabel et al. constructed an array of gene modules that are expressed commonly across multiple diseases. These gene modules were associated with certain functional characteristics as clarified by literature profiling [15, 29]. THP1r2*Mtb*-induced signature harbors nearly half (44/95) of the genes in the interferon-related module (M3.1) [6]. We found that 33 of these THP1r2*Mtb*-induced genes were also present in T*Mtb*-iNet, whereas only 11 of such THP1r2*Mtb*-induced genes were in T*Mtb*-iEx (*P* = 4.32 × 10^−6^) (Figure 7). Ingenuity pathway analysis also indicated that interferon signaling was enriched in T*Mtb*-iNet and THP1r2*Mtb*-induced signature with the highest significances, but not in T*Mtb*-iEx (−log_10_(*P* value) = 9.28 for T*Mtb*-iNet and –log_10_(*P* value) = 6.9 for THP1r2*Mtb*-induced). Taken together, our analyses validated that the network-based signature of T*Mtb*-iNet contained more interferon-related genes than the excluded signature of T*Mtb*-iEx and confirmed that our approach could select a network-based signature that retained the original signature's biological representation.

### 3.3. T*Mtb*-iNet Displays Equivalent Positive Correlation with PTB Patients but Higher Positive Correlation with Separated Cell Populations of PTB Patients Compared to THP1r2*Mtb*-Induced Signature or T*Mtb*-iEx

**Figure 8:**
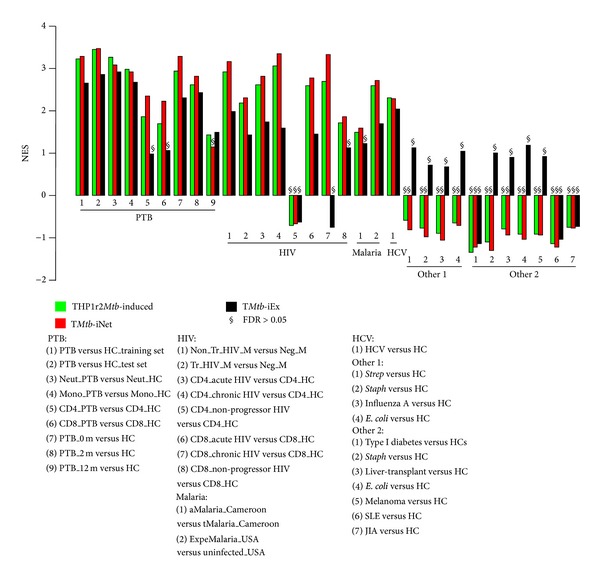
Plot of NES values of THP1r2*Mtb*-induced signature, T*Mtb*-iNet, and T*Mtb*-iEx from human macrophages against array data from human blood. NES values from GSEA with THP1r2*Mtb*-induced signature, T*Mtb*-iNet, or T*Mtb*-iEx against array data preranked against cognate healthy controls (HC) are plotted. Detailed explanations of the shortened descriptive phrases can be found in the footnotes of the relevant tables: Tables 1–3 for “PTB” group; Tables 5 and 6 for “HIV” group; Table 7 for “malaria” group; Table 8 for “HCV” group; Table 9 for “other 1” group; and Table 10 for “other 2” group.

We have previously indicated the high positive correlation of THP1r2*Mtb*-induced signature with a public transcriptional dataset on PTB patients [6]. We therefore examined whether the network-based signature of T*Mtb*-iNet still inherited the significant degree of positive correlation with PTB patients. As shown in Table 1 (also in Figure 8), like THP1r2*Mtb*-induced signature, T*Mtb*-iNet showed similar positive correlation with PTB more than with LTB and healthy controls (e.g., for the training set, PTB versus HC showed NES = 3.23 in THP1r2*Mtb*-induced signature, and NES = 3.30 in T*Mtb*-iNet). This was the case for any of the three datasets (i.e., London patient-based training set, London patient-based test set, and Cape Town patient-based validation set). In comparison, the correlation of T*Mtb*-iEx to PTB was lower (e.g., PTB versus HC showed NES = 2.66 in the training set). Our analysis indicated that the network-based signature of T*Mtb*-iNet, but not the excluded signature of T*Mtb*-iEx, was overall as expression-active as THP1r2*Mtb*-induced signature in the whole blood of PTB patients.

We then examined whether T*Mtb*-iNet also showed positive correlation with specific cell populations including neutrophils, monocytes, and CD4^+^ and CD8^+^ T cells from PTB patients. We found that, similar to THP1r2*Mtb*-induced signature, T*Mtb*-iNet showed positive and significant correlation with each of the four cell populations (Table 2 and Figure 8). However, with CD4^+^ and CD8^+^ T cells, T*Mtb*-iNet displayed higher positive correlation than THP1r2*Mtb*-induced signature (NES = 2.36 for T*Mtb*-iNet versus NES = 1.86 for THP1r2*Mtb*-induced signature in CD4^+^ T cells; NES = 2.23 for T*Mtb*-iNet versus NES = 1.70 for THP1r2*Mtb*-induced signature in CD8^+^ T cells). These higher correlations of T*Mtb*-iNet were specific to CD4^+^ and CD8^+^ T cells because, with neutrophils or monocytes, T*Mtb*-iNet did not show higher correlation than THP1r2*Mtb*-induced signature did. Thus, when compared to THP1r2*Mtb*-induced signature, T*Mtb*-iNet was more expression-active in CD4^+^ and CD8^+^ T cells, but not in neutrophils or in monocytes. T*Mtb*-iEx showed less correlation with neutrophils and monocytes than either T*Mtb*-iNet or THP1r2*Mtb*-induced signature, indicating that T*Mtb*-iEx was less expression-active in these two cell populations. More importantly, T*Mtb*-iEx displayed no correlation with CD4^+^ and CD8^+^ T cells, which indicated that T*Mtb*-iEx was not expression-active in T cells (Table 2). Taken together, our results indicated that the network-based signature of T*Mtb*-iNet provided equivalent correlation with PTB patients and higher correlation with CD4^+^ and CD8^+^ T cells, when compared to THP1r2*Mtb*-induced signature or the excluded signature of T*Mtb*-iEx.

### 3.4. T*Mtb*-iNet Decreases More than Either THP1r2*Mtb*-Induced Signature Or T*Mtb*-iEx during Treatment of PTB

Gene set enrichment analysis (GSEA) on the datasets from PTB patients receiving treatment indicated that T*Mtb*-iNet showed decreased, but still significant, positive correlation after two months of treatment (PTB_0 m versus HC with NES = 3.29 and FDR = 0 before treatment; PTB_2 m versus HC showed NES = 2.83 and FDR = 0 at 2 months after treatment), and the correlation of T*Mtb*-iNet became insignificant at 12 months after treatment (PTB_12 m versus HC with NES = 1.15 and FDR = 0.182) (Table 3 and Figure 8). By contrast, both THP1r2*Mtb*-induced signature and T*Mtb*-iEx still had significant positive correlation at 12 months after treatment, even though they showed decreasing correlation during the course of treatment (Table 3). Consistently, T*Mtb*-iNet showed lower negative correlation with PTB_2 m and PTB_12 m when compared to the pretherapy (PTB_0 m) (NES = −3.16 for T*Mtb*-iNet, NES = −2.96 for THP1r2*Mtb*-induced, and NES = −2.29 for T*Mtb*-iEx in PTB_12 m versus PTB_0 m) (Table 3). Similarly, T*Mtb*-iNet showed lower negative correlation than T*Mtb*-iEx with therapy of PTB at weeks 2, 4, and 26 after treatment compared to the pretherapy (PTB_wk0), but not at week 1 after treatment at another dataset (e.g., NES = −2.68 for T*Mtb*-iNet and NES = −2.38 for T*Mtb*-iEx in PTB_wk26 versus PTB_wk0) (Table 4 and Figure S2). THP1r2*Mtb*-induced signature showed the lowest negative correlation with PTB therapy at weeks 1, 2, and 4 but showed almost the same degree of negative correlation with T*Mtb*-iNet with PTB therapy at week 26 (e.g., NES = −2.71 for THP1r2*Mtb*-induced signature and NES = −2.68 for T*Mtb*-iNet in PTB_wk26 versus PTB_wk0) (Table 4 and Figure S2). These results collectively demonstrated that the network-based signature of T*Mtb*-iNet seemed to be more responsive to the therapy of PTB than the original THP1r2*Mtb*-induced signature or the excluded signature of T*Mtb*-iEx.

### 3.5. Correlation Analysis of T*Mtb*-iNet, THP1r2*Mtb*-Induced, and T*Mtb*-iEx Signatures to Several Other Infections and Inflammatory Conditions

HIV, malaria, and TB are the top infectious diseases imposing the heaviest burden on health care systems [30]. We therefore examined whether THP1r2*Mtb*-induced signature, T*Mtb*-iNet, and T*Mtb*-iEx were correlated with or well represented in transcriptome datasets from patients with HIV or malaria. We found that all the three signatures displayed general positive correlation in the transcriptome datasets of PBMCs from patients with HIV-1 infection (Table 5). Specifically, T*Mtb*-iNet displayed higher positive correlation than THP1r2*Mtb*-induced signature and T*Mtb*-iEx (e.g., NES = 3.16 for T*Mtb*-iNet versus NES = 2.93 for THP1r2*Mtb*-induced signature and NES = 1.98 for T*Mtb*-iEx) (Table 5 and Figure 8). Since HIV/TB coinfection imposes a severe death threat to patients and the underlying mechanism is the dysfunction of T cells [30], we then further applied the GSEA against transcriptome datasets from T cells (both CD4^+^ and CD8^+^) of patients with acute and chronic forms of HIV infection. All three signatures displayed general positive correlation in the T cell transcriptome datasets. By contrast, in the nonprogressor HIV group all the three signatures displayed no correlation with CD4^+^ T cells and lowest positive correlation with CD8^+^ T cells (Table 6). Notably, among the three signatures T*Mtb*-iNet showed the highest positive correlation with both CD4^+^ and CD8^+^ T cells from acute and chronic forms of HIV infection (e.g., in CD4_chronic HIV, NES = 3.35 for T*Mtb*-iNet versus NES = 3.06 for THP1r2*Mtb*-induced signature or NES = 1.59 for T*Mtb*-iEx) (Table 6 and Figure 8). Similarly, all the three signatures displayed positive correlation with malaria from either the natural malaria infection in Cameroon or the experimental challenge malaria in USA, and once again higher positive correlation was seen with T*Mtb*-iNet (e.g., in ExpeMalaria, NES = 2.73 for T*Mtb*-iNet versus NES = 2.59 for THP1r2*Mtb*-induced signature and NES = 1.71 for T*Mtb*-iEx) (Table 7 and Figure 8). In summary, blood samples from TB patients produced an interferon-related signature similar to those signatures seen in blood from patients with AIDS or malaria.

Since T*Mtb*-iNet showed correlation with non-TB conditions that also produce a similar interferon-related signature (AIDS and malaria here), we then further applied GSEA with T*Mtb*-iNet, along with THP1r2*Mtb*-induced signature and T*Mtb*-iEx, against other infections and inflammatory conditions [15, 21, 26]. All the three signatures showed strong positive correlation with datasets of PBMCs from chronic HCV-infected patients before drug treatment (Table 8) or during the therapy with pegylated interferon-2a (peginterferon-2a) and ribavirin, no matter whether the patients were African-American or Caucasian-American or were in any drug response category (i.e., marked, intermediate, or poor) (Table S4). No clear difference in correlation was observed between T*Mtb*-iNet and THP1r2*Mtb*-induced signature, although both of them showed higher positive correlation than T*Mtb*-iEx did (Tables 8 and S4 and Figures 8 and S3). However, all the three signatures showed no correlation with transcriptome datasets from patients with acute infections of* Streptococcus pneumonia*,* Staphylococcus aureus*, influenza A, or* E. coli *(Table 9) or from patients with inflammatory conditions of type I diabetes, liver transplant undergoing immunosuppressive therapy, metastatic melanoma, systemic lupus erythematosus, or systemic juvenile idiopathic arthritis (Table 10 and Figure 8). Thus, blood samples from patients with TB, AIDS, malaria, or hepatitis C displayed a common interferon-related signature that could be represented by our signatures, especially by the network-based signature of T*Mtb*-iNet.

## 4. Discussion

In this study, we combined our previously identified interferon-related THP1r2*Mtb*-induced signature with STRING protein-protein interaction data to generate a more refined version of T*Mtb*-iNet. The refined T*Mtb*-iNet still inherited key characteristics of THP1r2*Mtb*-induced signature. Promoter regions of genes in T*Mtb*-iNet were enriched with the TFBSs of ISRE, IRF-7, and IRF-1, and the whole T*Mtb*-iNet signature significantly overlapped with the interferon-inducible gene signature with PTB and interferon-related module (Figures 3, 6, and 7). Additionally, T*Mtb*-iNet showed strong positive correlation in PTB blood and its separated cell subpopulations, as well as patterns of decreasing positive correlation during the course of anti-TB therapy (Tables 1–4, Figure 8).

A complete set of protein-protein interactions comprises a summation of knowledge on functional modularity and network interconnectivity within cells [31]. Therefore, network-based interpretation of “omics” data should be more rational and biology oriented than one based solely on transcriptomics [10–12, 32, 33]. Here we identified characteristic protein-protein connections within the THP1r2*Mtb*-induced profile with the involvement of a third factor (hub) (Figures 2 and 3, Figure S1). By integration of protein-protein interaction data, we refined a subset of genes from the interferon-related THP1r2*Mtb*-induced transcriptome signature [6] to obtain T*Mtb*-iNet and discarded the rest into T*Mtb*-iEx (Figure 4 and Table S3). Compared with THP1r2*Mtb*-induced signature or T*Mtb*-iEx, T*Mtb*-iNet consistently enriched interferon signaling and interferon-related TFBSs of ISRE, IRF-1, and IRF-7 in the promoter regions of its genes (Figure 3), as well as harboring more interferon-related genes (Figures 6 and 7). In addition, T*Mtb*-iNet showed greater positive correlation with the separated cells from PTB patients (neutrophils, monocytes, and CD4^+^ and CD8^+^ T cells) (Table 2) and specifically displayed a decreasing pattern of positive correlation during therapy of PTB (Table 3). All these results indicated the reliability of the hub-based network approach for identifying a functionally enriched signature.

A key finding was that there exists a universal core of functionally associated host responses irrespective of immune cell type. Transcriptional responses of immune and adaptive immune cells during human* M. tb* infection have been studied by others. After migrating to tissues (e.g., lung), monocytes can differentiate into macrophages and dendritic cells which are major phagocytes that engulf* M. tb* and induce adaptive immunity [34, 35]. CD4^+^ and CD8^+^ T cells are both important adaptive immune cells in TB and dysfunction of either significantly abrogates control of TB infection [34]. Neutrophils can also be a prominent cell type infected by* M. tb* [36]. Circulating monocytes undergo functional and phenotypic changes in TB patients, although the presence of different subtypes of monocytes in peripheral blood may have reverse implications for TB control at sites of infection [37, 38]. As a refined signature of function, T*Mtb*-iNet showed a higher degree of positive correlation with all the four separated peripheral blood cell populations of PTB patients (*i.e.,* neutrophils, monocytes, and CD4^+^ and CD8^+^ T cells) with the highest positive correlation with neutrophils (Table 2, Figure 8). Thus, a common host response existed among these immune cells in PTB patients, irrespective of cell type or* in vitro* (i.e., THP-1) or* in vivo* (i.e., PTB) conditions. This was consistent with other reports showing that immune cells often exhibit a core gene expression profile when exposed to various microorganisms [39–42]. Another key finding was the decreased pattern of correlation of T*Mtb*-iNet during clinical therapy of PTB patients (Table 3), which suggested that this set of genes (T*Mtb*-iNet) or the biological process behind their activity (probably an interferon-related process) was strongly involved in the generation/treatment of PTB as also reported by others [2, 3, 43, 44]. The interferon-based nature of the T*Mtb*-iNet and the THP1r2*Mtb*-induced signatures was indirectly validated by the strong positive correlation with the therapy of chronic hepatitis C; type 1 interferon-related processes were inevitably activated in these patients because they were treated with peginterferon-2a (Table S4, Figure S3).

Interestingly, T*Mtb*-iNet also displayed stronger positive correlation with AIDS and malaria, as well as with hepatitis C (Tables 5–8, Figure 8), but not with several other acute infections or inflammatory conditions (Tables 9 and 10, Figure 8). This indicated that a similar host response (an interferon-related process being most likely in this case) is shared when a host is fighting/adjusting against these three pathogens of diverse phyla. Coinfection with HIV is known to increase latent TB reactivation about 20-fold [30]. T cells are vital in adaptive control of* M. tb* infection [45]; however, HIV infection can gradually deplete CD4^+^ T cells, some of which can be* M. tb*-specific [30] and CD4^+^ T cell depletion is a key factor contributing to latent TB reactivation [30, 46]. Other changes in host cells caused by HIV can also facilitate* M. tb* survival, such as disruption of bactericidal activities of macrophages [47, 48] and deregulation of chemotaxis [49]. GSEA analysis (Tables 1–4, Figure 8) and transcriptome analysis on PTB patients confirmed that interferon-related processes are dynamically regulated in the pathogenesis/treatment of PTB [2]. We demonstrated a positive correlation of T*Mtb*-iNet with CD4^+^ and CD8^+^ T cells from PTB patients or AIDS patients (acute and chronic forms of HIV infection), forming a transcriptome bridge of similarity (i.e., an interferon-related process) between these two diseases [23]. However, the functional significance of such similarity remains elusive in TB/HIV coinfection patients. Detailed transcriptional profiling with high-throughput analyses is needed to unravel the sophisticated mutual correlation and potential for clinical utility in TB, HIV, and TB/HIV coinfection patients. A recent network-based transcriptome study based on mouse models noted strong overlap between genes regulated during cerebral malaria and genes regulated during* M. tb* infections [50]. These observations indicate caution before using transcriptional signatures alone for TB diagnosis or prognosis.

The observation that T*Mtb*-iNet represented a transcriptional response related to patients with AIDS, malaria, and HCV (Tables 5–8) might suggest that the microorganism* M. tb* expressed a molecular pattern which was also expressed by the other disease agents during infections. Innate immune recognitions of HIV and HCV primarily involve sensing of nucleic acids. A DNA-containing protein complex from* Plasmodium falciparum*, the causative agent of malaria, is also known to be the major trigger of the innate immune response [51]. In a study based on mouse macrophages it was shown that* M. tb* activates a nucleic acid sensing pathway [52]. Thus, nucleic acids of* M. tb*, malaria, HIV, and HCV might produce the common transcriptional response in immune cells represented by T*Mtb*-iNet. It is of note that T*Mtb*-iNet does not reflect a generic interferon-related signature. Other pathogens, such as* S. pneumoniae* [53] and influenza A [54], also trigger nucleic acid-dependent immune responses, but they failed to induce a T*Mtb*-iNet-related transcriptional signature (Tables 9 and 10).

In summary, we derived a refined network signature (T*Mtb*-iNet) from the original transcriptional signature (THP1r2*Mtb*-induced) based on their directions among themselves or through a group of hubs. The refined signature T*Mtb*-iNet was a highly connected signature induced by* M. tb* infections* in vitro*. It showed positive correlation with clinical TB. We believe that the gene products of T*Mtb*-iNet, especially those gene products with higher degrees of interaction, as well as the connecting hubs, are major regulators of immune responses to TB. The shared correlation of T*Mtb*-iNet with other important infectious diseases deserves the attention of investigators involved in developing transcriptome-based TB diagnostic or prognostic tests.

## Supplementary Material

Supplementary Material contains three figures and five tables (FIGURES S1-S3 and TABLES S1-S5). FIGURE S1: General feature of THP1r2*Mtb*-iNet[*i*] and its cognate hubs.; FIGURE S2: Correlation of THP1r2*Mtb*-induced, T*Mtb*-iNet, and T*Mtb*-Ex during therapy of PTB; FIGURE S3: Correlation of THP1r2*Mtb*-induced, T*Mtb*-iNet, and T*Mtb*-Ex during therapy of chronic hepatitis C; Table S1: General features of THP1r2*Mtb*-iNet[*i*]; TABLE S2: TFBS profiling of THP1r2*Mtb*-iNet[*i*]; TABLE S3: Relative expression levels of T*Mtb*-iNet and its cognate hubs in THP-1 cells; TABLE S4: GSEA using transcriptome data of PBMCs from chronic HCV-infected patients undergoing therapy; TABLE S5. Gene lists from KEGG analysis in THP1r2*Mtb*-induced, T*Mtb*-iNet, and T*Mtb*-iEx.

## Figures and Tables

**Table 1:** GSEA using transcriptome data of whole blood from human PTB patients.

Cohort^1^	Group comparison^2^	THP1r2*Mtb*-induced			T*Mtb*-iNet			T*Mtb*-iEx		
		NES^3^	FDR^4^	Correlation^4^	NES^3^	FDR^4^	Correlation^4^	NES^3^	FDR^4^	Correlation^4^
Training set	PTB versus HC	3.23	0.000	Positive	3.30	0.000	Positive	2.66	0.000	Positive
	PTB versus LTB	3.00	0.000	Positive	3.01	0.000	Positive	2.53	0.000	Positive
	LTB versus HC	−1.22	0.071	Null	−0.91	0.902	Null	−1.37	0.062	Null

Test set	PTB versus HC	3.46	0.000	Positive	3.48	0.000	Positive	2.86	0.000	Positive
	PTB versus LTB	3.12	0.000	Positive	3.23	0.000	Positive	2.51	0.000	Positive
	LTB versus HC	2.37	0.000	Positive	2.25	0.000	Positive	2.11	0.000	Positive

Validation set	PTB versus LTB	3.27	0.000	Positive	3.28	0.000	Positive	2.59	0.000	Positive

^1^Training set and test set: all donors were from London, UK; PTB: pulmonary TB; LTB: latent TB; HC: healthy controls; validation set: all donors were from Cape Town, South Africa [2].

^2^Group-group contrasted and ranked by LIMMA-based method [27, 28].

^3^(+) NES for positive correlation, (−) NES for negative correlation.

^4^A FDR of 0.05 or lower was regarded as statistically significant for NES (“positive” or “negative”), otherwise “Null.”

**Table 2:** GSEA using transcriptome data of separated cell populations from human PTB patients.

Group comparison^1^	THP1r2*Mtb*-induced			T*Mtb*-iNet			T*Mtb*-iEx		
	NES^2^	FDR^3^	Correlation^3^	NES^2^	FDR^3^	Correlation^3^	NES^2^	FDR^3^	Correlation^3^
Neut_PTB versus Neut_HC	3.27	0.000	Positive	3.08	0.000	Positive	2.92	0.000	Positive
Mono_PTB versus Mono_HC	2.98	0.000	Positive	2.93	0.000	Positive	2.67	0.000	Positive
CD4_PTB versus CD4_HC	1.86	0.000	Positive	2.36	0.000	Positive	0.99	0.484	Null
CD8_PTB versus CD8_HC	1.70	0.000	Positive	2.23	0.000	Positive	1.07	0.238	Null

^1^Neut: purified neutrophils; Mono: purified monocytes; CD4: purified CD4^+^ T cells; CD8: purified CD8^+^ T cells [2].

^2,3^The same as Table 1.

**Table 3:** GSEA using transcriptome data of whole blood from human PTB patients undergoing clinical therapy.

Group comparison^1^	THP1r2*Mtb*-induced			T*Mtb*-iNet			T*Mtb*-iEx		
	NES^2^	FDR^3^	Correlation^3^	NES^2^	FDR^3^	Correlation^3^	NES^2^	FDR^3^	Correlation^3^
PTB_0m versus HC	2.95	0.000	Positive	3.29	0.000	Positive	2.31	0.000	Positive
PTB_2m versus HC	2.62	0.000	Positive	2.83	0.000	Positive	2.43	0.000	Positive
PTB_12m versus HC	1.43	0.000	Positive	1.15	0.182	Null	1.49	0.005	Positive
PTB_2m versus PTB_0m	−2.08	0.000	Negative	−2.36	0.000	Negative	−1.53	0.000	Negative
PTB_12m versus PTB_0m	−2.96	0.000	Negative	−3.16	0.000	Negative	−2.29	0.000	Negative

^1^PTB_0m: PTB patients before drug treatment; PTB_2m: 2 months after initiation of drug treatment; PTB_12m: 12 months after initiation of drug treatment [2].

^2,3^The same as Table 1.

**Table 4:** GSEA using another transcriptome data of whole blood from human PTB patients undergoing clinical therapy.

Group comparison^1^	THP1r2*Mtb*-induced			T*Mtb*-iNet			T*Mtb*-iEx		
	NES^2^	FDR^3^	Correlation^3^	NES^2^	FDR^3^	Correlation^3^	NES^2^	FDR^3^	Correlation^3^
PTB_wk1 versus PTB_wk0	−1.96	0.000	Negative	−1.82	0.000	Negative	−1.87	0.000	Negative
PTB_wk2 versus PTB_wk0	−2.04	0.000	Negative	−2.00	0.000	Negative	−1.80	0.000	Negative
PTB_wk4 versus PTB_wk0	−2.54	0.000	Negative	−2.37	0.000	Negative	−2.31	0.000	Negative
PTB_wk26 versus PTB_wk0	−2.71	0.000	Negative	−2.68	0.000	Negative	−2.38	0.000	Negative

^1^PTB_wk0: PTB patients before drug treatment; PTB_wk1: 1 week after initiation of drug treatment; PTB_wk2: 2 weeks after initiation of drug treatment; PTB_wk4: 4 weeks after initiation of drug treatment; PTB_wk26: 26 weeks after initiation of drug treatment [20].

^2,3^The same as Table 1.

**Table 5:** GSEA using transcriptome data of PBMCs from AIDS mothers with infants.

Group comparison^1^	THP1r2*Mtb*-induced			T*Mtb*-iNet			T*Mtb*-iEx		
	NES^2^	FDR^3^	Correlation^3^	NES^2^	FDR^3^	Correlation^3^	NES^2^	FDR^3^	Correlation^3^
Non_Tr_HIV_M versus Neg_M	2.93	0.000	Positive	3.16	0.000	Positive	1.98	0.000	Positive
Tr_HIV_M versus Neg_M	2.20	0.000	Positive	2.32	0.000	Positive	1.44	0.011	Positive

^1^Non_Tr_HIV_M: HIV-1 positive mothers who did not transmit the virus to their infants; Tr_HIV_M: HIV-1 positive mothers who perinatally transmitted the virus to their infants; Neg_M: HIV negative mothers.

^2,3^The same as Table 1.

**Table 6:** GSEA using transcriptome data of CD4^+^ or CD8^+^ T cells from AIDS patients.

Group comparison^1^	THP1r2*Mtb*-induced			T*Mtb*-iNet			T*Mtb*-iEx		
	NES^2^	FDR^3^	Relevance^3^	NES^2^	FDR^3^	Correlation^3^	NES^2^	FDR^3^	Correlation^3^
CD4_acute HIV versus CD4_HC	2.61	0.000	Positive	2.82	0.000	Positive	1.75	0.000	Positive
CD4_chronic HIV versus CD4_HC	3.06	0.000	Positive	3.35	0.000	Positive	1.59	0.006	Positive
CD4_nonprogressor HIV versus CD4_HC	−0.70	1.000	Null	−0.66	1.000	Null	−0.62	1.000	Null
CD8_acute HIV versus CD8_HC	2.59	0.000	Positive	2.78	0.000	Positive	1.46	0.013	Positive
CD8_chronic HIV versus CD8_HC	2.70	0.000	Positive	3.34	0.000	Positive	−0.75	0.912	Null
CD8_nonprogressor HIV versus CD8_HC	1.73	0.000	Positive	1.87	0.000	Positive	1.13	0.127	Null

^1^Acute HIV: early infection; chronic HIV: chronic, no treatment; nonprogressor: long-term nonprogressor.

^2,3^The same as Table 1.

**Table 7:** GSEA using transcriptome data of PBMCs from malaria patients.

Group comparison^1^	THP1r2*Mtb*-induced			T*Mtb*-iNet			T*Mtb*-iEx		
	NES^2^	FDR^3^	Correlation^3^	NES^2^	FDR^3^	Correlation^3^	NES^2^	FDR^3^	Correlation^3^
aMalaria_Cameroon versus tMalaria_Cameroon	1.50	0.005	Positive	1.60	0.003	Positive	1.23	0.100	Null
ExpeMalaria_USA versus Uninfected_USA	2.59	0.000	Positive	2.73	0.000	Positive	1.71	0.001	Positive

^1^aMalaria_Cameroon: acute malaria patients from Cameroon; tMalaria_Cameroon: treated malaria patients from Cameroon; ExpeMalaria_USA: Experimental malaria donors from USA; Uninfected_USA: baseline uninfected donors from USA.

^2,3^The same as Table 1.

**Table 8:** GSEA using transcriptome data of PBMCs from patients with chronic HCV infection.

Group comparison^1^	THP1r2*Mtb*-induced			T*Mtb*-iNet			T*Mtb*-iEx		
	NES^2^	FDR^3^	Correlation^3^	NES^2^	FDR^3^	Correlation^3^	NES^2^	FDR^3^	Correlation^3^
HCV versus HC	2.32	0.000	Positive	2.30	0.000	Positive	2.05	0.000	Positive

^1^HCV: treatment-naïve chronic HCV-infected patients. HC: healthy controls.

^2,3^The same as Table 1.

**Table 9:** GSEA using transcriptome data of PBMCs from human patients with acute infections.

Group comparison^1^	THP1r2*Mtb*-induced			T*Mtb*-iNet			T*Mtb*-iEx		
	NES^2^	FDR^3^	Correlation^3^	NES^2^	FDR^3^	Correlation^3^	NES^2^	FDR^3^	Correlation^3^
*Strep* versus HC	−0.58	0.996	Null	−0.81	1.000	Null	1.14	0.225	Null
*Staph* versus HC	−0.77	0.871	Null	−0.97	1.000	Null	0.72	0.945	Null
Influenza A versus HC	−0.88	0.688	Null	−1.05	1.000	Null	0.68	0.952	Null
*E. coli* versus HC	−0.65	0.963	Null	−0.70	1.000	Null	1.05	0.364	Null

^1^ *Strep: Streptococcus pneumonia* infection; *Staph: Staphylococcus aureus* infection.

^2,3^The same as Table 1.

**Table 10:** GSEA using transcriptome data of PBMCs from human patients with other inflammatory or pathological conditions.

Group comparison^1^	THP1r2*Mtb*-induced			T*Mtb*-iNet			T*Mtb*-iEx		
	NES^2^	FDR^3^	Correlation^3^	NES^2^	FDR^3^	Correlation^3^	NES^2^	FDR^3^	Correlation^3^
Type I diabetes versus HC	−1.34	0.112	Null	−1.21	0.329	Null	−1.13	0.336	Null
*Staph* versus HC	−1.09	0.305	Null	−1.29	0.334	Null	1.00	0.441	Null
Liver-transplant versus HC	−0.79	0.892	Null	−0.92	1.000	Null	0.90	0.647	Null
*E. coli* versus HC	−0.91	0.646	Null	−1.02	1.000	Null	1.20	0.156	Null
Melanoma versus HC	−0.91	0.641	Null	−0.92	1.000	Null	0.92	0.622	Null
SLE versus HC	−1.14	0.396	Null	−1.22	0.540	Null	−1.04	0.490	Null
JIA versus HC	−0.74	1.000	Null	−0.76	1.000	Null	−0.73	0.908	Null

^1^ *Staph*: *Staphylococcus aureus* infection; liver-transplant: liver-transplant undergoing immunosuppressive therapy; melanoma: metastatic melanoma; SLE: systemic lupus erythematosus; JIA: systemic juvenile idiopathic arthritis.

^2,3^The same as Table 1.
